# A novel procedure for absolute real-time quantification of gene expression patterns

**DOI:** 10.1186/1746-4811-8-9

**Published:** 2012-03-09

**Authors:** Yingqing Lu, Lulu Xie, Jiani Chen

**Affiliations:** 1 State Key Laboratory of Systematic and Evolutionary Botany, Institute of Botany, Chinese Academy of Sciences, 20 Nan Xin Cun, Beijing 100093, China; 2 Graduate School of the Chinese Academy of Sciences, Beijing 100049, China

**Keywords:** Real-time qPCR, Transcript number, cDNA quantitation, Gene expression pattern

## Abstract

**Background:**

Temporal and tissue-specific patterns of gene expression play important roles in functionality of a biological system. Real-time quantitative polymerase chain reaction (qPCR) technique has been widely applied to single gene expressions, but its potential has not been fully released as most results have been obtained as fold changes relative to control conditions. Absolute quantification of transcripts as an alternative method has yet to gain popularity because of unresolved issues.

**Results:**

We propose a solution here with a novel procedure, which may accurately quantify the total cDNA conventionally prepared from a biological sample at the resolution of ~70 pg/μl, and reliably estimate the absolute numbers of transcripts in a picogram of cDNA. In comparison to the relative quantification, cDNA-based absolute (CBA) qPCR method is found to be more sensitive to gene expression variations caused by factors such as developmental and environmental variations. If the number of target transcript copies is further normalized by reference transcripts, cell-level variation pattern of the target gene expression may also be detectable during a developmental process, as observed here in cases across species (*Ipomoea purpurea, Nicotiana benthamiana*) and tissues (petals and leaves).

**Conclusion:**

By allowing direct comparisons of results across experiments, the new procedure opens a window to make inferences of gene expression patterns across a broad spectrum of living systems and tissues. Such comparisons are urgently needed for biological interpretations of gene expression variations in diverse cells.

## Background

Being a key stage of functional realization of genome, gene expression has been increasingly quested for more details in various investigations [1-4]. Relative to still costly implementations of omics approaches, real-time quantitative polymerase chain reaction (qPCR) technique remains a top choice for comparison of gene expressions in cases of a small gene number but variable sample sizes because of the sensitivity of florophors, the efficiency of PCR [5], and the relatively low cost. Albeit having widespread usages in laboratories [6], qPCR may have some difficulties in interpretations of its results since gene expressions have been estimated as fold changes [7], which may be hard to compare across tissues and experiments without a common basis.

Depending on the appropriate internal control (reference) genes, the relative qPCR method estimates fold change of expression difference between target and reference genes relative to a control condition through 2^-ΔΔCt ^calculation [7,8]. As a priori for the relative qPCR, the choice of reference genes needs to be experimentally validated [8]. Even with the priori met, different reference genes could be chosen across tissues or among species for the same biological process [e.g. fruit development, [9] vs. [10]], making a direct comparison of the results troublesome, while complexity of biological systems makes it unlikely to find a universal gene expression for the purpose of broad-scale comparisons. Attempts have been made to add more reference genes in the estimation to increase its reliability; still, the practice does not improve the power of interpretation, nor has it the theoretical basis to do so.

Besides fold changes, gene expressions can also be evaluated for a known quantity of cDNA [11] or RNA [12,13] as previously tried. In comparison to RNA, cDNAs are more stable during dilution procedure as observed in environmental samples [14]. Because only DNA is directly involved in PCR, RNA has to be transcribed into cDNA to be detectable in qPCR. This step, however, is prone to unknown degrees of quantification errors, as reverse transcription among samples might occur in various efficiencies. Relatively speaking, cDNA, if quantified accurately, is more appropriate than RNA to be a comparison basis for qPCR results.

There are biological implications for taking cDNA as a comparison basis for gene expression. First, not all mRNA species have poly-A at the 3' end, and these without A-ending are excluded outside the cDNA pool after the conventional first-strand synthesis. Fortunately, most transcripts are included in the synthesis, and cDNA synthesized is largely representative of the mRNA in the total RNA for a sample. Second, regardless of the relatively small proportion of the mRNA in the total RNA in quantity, a strong correlation has been observed between mRNA and the total RNA [15] and excessive rRNA can cause a growth defect [16]. These results suggest that the proportions of various RNAs may be relatively stable for a normally functional genome, which implies that the overall quantity of mRNA for a given amount of tissue may be indicative of the average genomic expression. Since a significant correlation is expected between mRNA and cDNA, a quantity of cDNA may thus be taken as a proxy of the whole genomic expression in a certain number of cells at the time of sampling. Typically, each species of transcript constitutes a small fraction of the whole cDNA pool, its change may be detected against a given quantity of cDNA when the latter is in a steady-state condition (e.g., in mature tissues). Changes in the absolute transcript number can therefore be readily interpreted against this common background and comparable across tissues and experiments.

A challenge to the above argument, however, comes when the genomic expression is not stable at the cellular level, as during a developmental process or an environmental stimulation. In these cases, the quantity of the total cDNA is no longer a fixed proxy for a given amount of cells, which hence invalidates a comparison between samples. A remedy for this lack of comparison is recommended here - taking the expression of housekeeping genes as the basis of normalization. These genes have the assumed property of stable transcription due to their conserved functional roles in cells. When the assumption roughly holds, their expressions may be taken as an internal control for the fluctuating genomic expression at the cellular level. If target genes are not of housekeeping type, their gene expressions normalized by these of housekeeping genes may in theory capture the pattern of the target transcript variation over a dynamic process.

While cDNA-based comparison of gene expression is logically sound, quantification of a conventionally prepared cDNA can be problematic, particularly in small tissues. The application of enzymes, particularly DNase, may severely reduce sample yield, often not practical for small samples. A typical cDNA sample after RNase treatment (which is much milder than DNase in yield loss) is a mixture of single stranded (ss-) cDNA, carried-over DNA from the RNA extraction, and unincorporated oligo dT in various amounts. Although Rhinn et al. [17] has proposed a direct quantification of cDNA without RNase treatment but using different sensitivities of Oligreen-emitted fluorescence between ss-DNA and RNA at 80°C, a large contribution of carried-over DNA to the fluorescent detection results in a low resolution of the method for a conventionally prepared cDNA sample in our previous trials. A need for a reliable quantification of conventionally prepared cDNA is real.

Here, we describe a well-tested method of cDNA quantitation in our laboratory using two fluorescent dyes - SYBR Green II and Picogreen [18], and show how the absolute number of transcript copies in biological samples can be reliably estimated with a good resolution. We first provide the theoretical basis for the feasibility of the protocol, then discuss in some details the pros and cons of the absolute qPCR method versus those of the relative qPCR approach by examples. Along with the samples from developing petals of *Ipomoea purpurea *(the common morning glory) and developing leaves of both *I. purpurea *and *Nicotiana benthamiana*, we show the applicability of the novel procedure to wide biological systems.

## Results and discussion

### Feasibility of using SYBR Green II and Picogreen in cDNA quantitation

**Figure 1:**
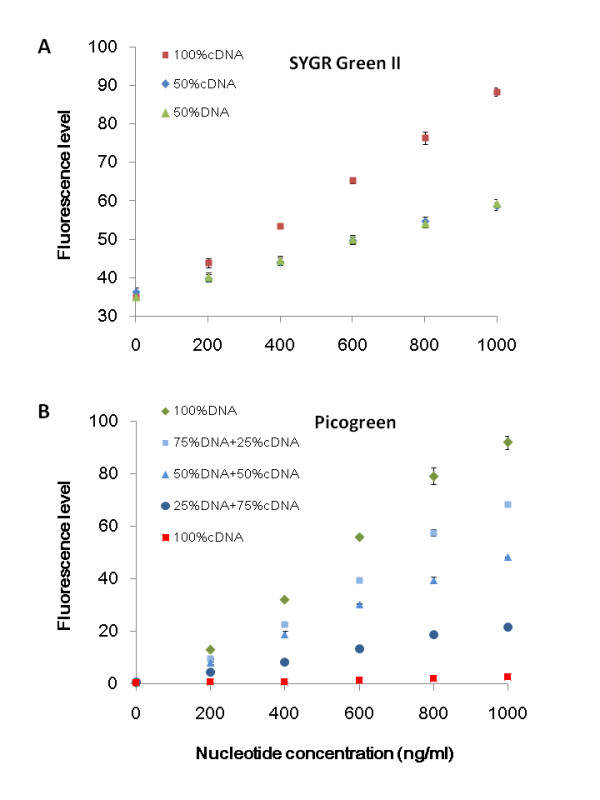
**Features of two fluorescent dyes**. (**A**) Additive fluorescent emissions of SYBR Green II. For each concentration level, three samples were prepared individually in triplets: 50% DNA (50% purified DNA + 50% pure water), 50% cDNA (50% purified cDNA + 50% pure water), and 50% cDNA + 50% DNA. Each was mixed in equal quantity with the dye solution (10 μl SYBR Green II buffer +10 μl sample) at 25°Cfor 5 min before taking measurements. Standard errors are indicated by black bars. (**B**) Fluorescent emissions of Picogreen in mixed solutions. Pure cDNA (100% cDNA), DNA (100% DNA), and their mixtures in different proportions were prepared in triplets, and mixed with Picogreen solution (10 μl Picogreen buffer +10 μl sample) before measurements were taken. Bar indicates the standard error of each mean.

The first part of our procedure is to accurately quantify cDNA in a conventionally prepared sample. SYBR Green II was previously shown via a fluorometer to bind to DNA and RNA additively in TE solution with 0.5% sodium desoxycholate [19]. It is desirable for us to know if the same behavior holds for mixed DNA and cDNA under the setting of a qPCR machine. We observed the additivity in our purified samples (Figure 1A), and further found that Picogreen can bind to DNA predictably in the presence of a wide range of cDNA concentrations (Figure 1B). Hence, the difference of the two dye estimates can form the basis for cDNA sample quantitation (Table 1), as shown in the case of anthocyanin pathway gene - *bh2c *(EU032620) at the locus of *IpbHLH2 *expressed in *I. purpurea *corollas on the common morning glory growing in the field.

**Table 1:** Quantitation of cDNA samples

Sample name*	SYBR Green II (pg/μl)	Picogreen (pg/μl)	cDNA concentration (pg/μl)
2008-9-23 cDNAs	198.62 ± 16.69	16.59 ± 2.50	182.03
2008-9-24 cDNAs	220.33 ± 45.29	14.61 ± 0.68	205.72
2008-9-25 cDNAs	223.01 ± 46.84	11.36 ± 0.30	211.66
2008-9-26 cDNAs	142.33 ± 18.37	10.04 ± 0.65	132.29
2008-9-27 cDNAs	221.67 ± 11.32	10.98 ± 0.67	210.69

* Samples were named after the collection dates. Each sample of *Ipomoea purpurea *corolla was diluted 50×, and then measured with two fluorescent dyes, separately. Columns with two numbers show the mean and the standard error based on triplets.

To verify that the self-made cDNA standard was in good quality, known quantities of purified DNA, RNA and cDNAs were compared, and the quality of cDNA was confirmed. The sensitivity of Picogreen dye allowed a dependable detection of residual gDNA with little interference from cDNAs or RNAs (Additional file 1: Figure S1). In the case of SYBR Green II detection, as little as 50-70 pg/μl single stranded nucleotides were measurable. A clear separation of cDNA and DNA can be detected in a mixed solution from 200 to 800 pg/μl. Further, to evaluate a possible effect of oligo dT in inflating the total cDNA estimation, we performed an experiment that started with the maximum presence of the primer (2 μl of 100 μm), and followed the procedures from cDNA synthesis (without the initial RNA included) to SYBR Green II quantification, and observed no significant signals in all cases (n = 3, each measured 3 times). We concluded that the conventional amount of oligo primers did not affect the accuracy of the cDNA quantification.

### Estimation of absolute transcript numbers of target genes with SYBR green I

In the second portion of the procedure, SYBR Green I is used in the qPCR reactions because of its higher precision and a lower coefficient of variation than those of TagMan and probe hybridization [20]. After 40 PCR cycles, one copy of a transcript may be represented as thousands of fluorescent fragments and become detectable [21]. In our samples, we found as few as one copy of a given transcript per fg cDNA.

**Figure 2:**
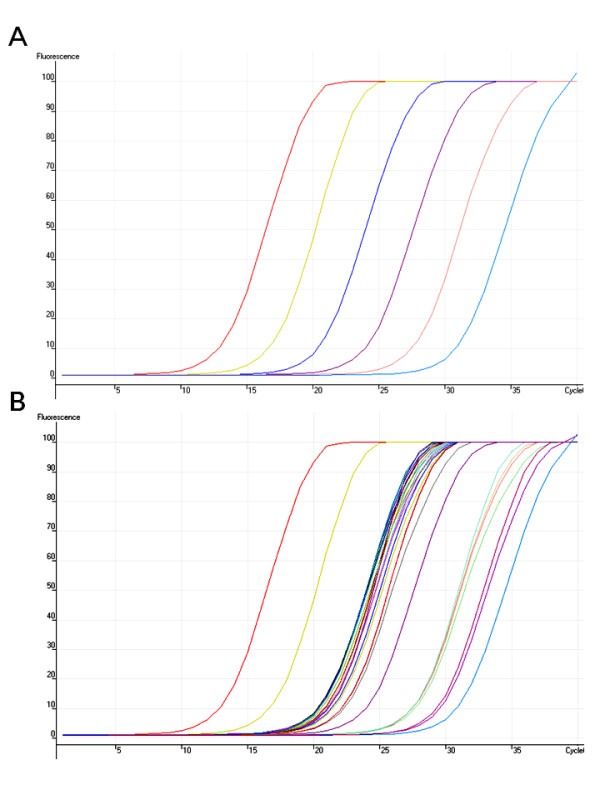
**Amplifications of *bH2c *transcript in floral samples**. Petals of the common morning glory were taken 12 hours before floral opening on five consecutive days (n = 5) on the same plant (III6D). Each sample was represented by three repeats in the same qPCR run. (**A**) The target standard curve; (**B**) The unknown samples amplified along with the standard in the same run. The fluorescent level of SYBR Green I was measured at 510 nm.

An example was given again for *bh2c *allele of *IpbHLH2 *expressed in corollas. As shown in Table 1, the cDNA concentration was determined prior to the qPCR detection. The target transcript had been cloned previously [22], and was quantified by Picogreen with known quantities of λDNA, and then taken as the standard in the later qPCR amplification with SYBR Green I (Figure 2). The standard provided the linear series (log scale) for the inference of transcript quantities of the same gene in unknown samples (Table 2). As both standard and unknown samples were placed in the same run, experimental errors may be reduced to minimum. The transcript quantities estimated were expressed in pg/μl and the amount of cDNA added into each reaction was in the range of 1-10 ng/μl. The absolute number of transcript copies was calculated in the formula below using the Avogadro's constant:

**Table 2:** Quantitation of absolute numbers of *bh2c *transcript copies in the corolla samples of *Ipomoea purpurea*

Sample name	Type	Ct	Given concentration (copies/μl)	Calculated Concentration (copies/μl)	cDNAs concentration* (pg/μl)	Transcript copies/fg cDNAs**
bh2c-1	Standard	6.67	117363.39	128533.38		
bh2c-2	Standard	10.21	11736.33	10758.19		
bh2c-3	Standard	13.24	1173.63	1284.77		
bh2c-4	Standard	16.83	117.36	104.04		
bh2c-5	Standard	19.93	11.74	11.80		
bh2c-6	Standard	23.4	1.17	1.04		
bh2c-7	Standard	26.31	0.117	0.135		
bh2c 2008-9-23	Unknown	13.22 ± 0.03		1302.40 ± 24.43	9101.52	143.10 ± 2.68
bh2c 2008-9-24	Unknown	20.02 ± 0.04		11.14 ± 0.30	10285.83	1.08 ± 0.03
bh2c 2008-9-25	Unknown	14.08 ± 0.02		714.67 ± 8.61	10582.80	67.53 ± 0.81
bh2c 2008-9-26	Unknown	13.95 ± 0.01		784.27 ± 6.37	6614.55	118.57 ± 0.96
bh2c 2008-9-27	Unknown	13.27 ± 0.01		1261.69 ± 5.28	10534.68	119.77 ± 0.50

*The concentrations of cDNAs (pg/μl) were derived from Table 1 after taking 50 × factor into account.

** The copy number was estimated via equation (1). Standard errors (± se) are based on triplet measurements.

(1) $$trancript\;copies\;=\frac{6.022141\times 10^{23}\;\times transcript\;quantity}{MW}\;$$

To reduce other sources of errors, we extracted the total RNAs with the Trizol reagent by reason of its robustness and reproducibility [23]. Attention was also paid to the linear range of fluorescent emissions. For example, although the range of linearity between the fluorescent emission of SYBR Green II and the nucleotide quantity has been shown between 10 and 1000 pg/μl [19], the most reliable estimates fall in the middle range as SYBR Green II has a low level of intrinsic fluorescence (Figure 1B). As a comparison, the precision of Picogreen (25 fg DNA/μl) is a magnitude higher, thus imposing little effect on the overall accuracy of the method. The precision of the cDNA quantitation is mostly set by SYBR Green II.

Moreover, primer design may be optimized to flank an intron to eliminate a potential contribution of gDNA to the final product of PCR amplification. As gDNA typically constitutes less than 10% of the cDNA sample (Table 1), the chance for the target gene from the gDNA source to be amplified in the qPCR is rather slim. Although no such amplifications were observed in our reactions, checking the melting curve of each run and sequencing the PCR product from time to time help judge whether or not such problem has occurred. Experimental errors may also come from using suboptimal amplification conditions and arranging samples in different experimental runs, which may be avoided with a careful plan.

### Gene expressions of *ACTIN *and *GAPDH *shift significantly across floral developmental stages and between species

To evaluate how expression patterns of commonly applied reference genes fare in the cDNA based absolute (CBA) procedure and the relative quantification method, we cloned reference genes from cDNA samples in *I. purpurea *(*IpACTIN4*: JN882352 and *IpGAPDH2*: JN882353) and *N. benthamiana *(*NbGAPDH*: JQ256517 and *NbACTIN*: JQ256516). *IpACTIN4 *was a homolog of gene *actin 4 *(accession number: HM802138) in *Ipomoea nil *[24], judging from the similarity of 99.9% between their coding regions. *IpGAPDH2 *is about 99% similar to *InGAPDH2 *(accession number: AB449345) expressed in *I. nil *[25]. Similarly, *NbGAPDH *and *NbACTIN *of *N. benthamiana *are homologs of *gapdh *(DQ682459) and *actin *(AY158612) in *N. tabacum*, respectively. They were used as house-keeping genes without further search as their involvement in this study is mainly for showing the validity of the new approach rather than taking as the optimized reference genes.

During the petal development of *I. purpurea*, expressions of *IpACTIN4 *and *IpGAPDH2 *were profiled in two cases (60 and 90 hours before flowering (HBF)), both of which showed significant variations (Table 3). While holding the floral development at the same stage (36 HBF), petals sampled on four dates again displayed large variances of the gene expressions (Table 3). Even for samples taken at hourly interval within the same day, considerable variances of the reference gene expressions appeared, as in the cases of SXSX2-2 and SXSX2-8 (Table 3). In a statistical test combining the latter two cases (n = 72), we observed significant effects of specific gene locus (gene), genotype, and time of sampling on transcript copy number (Additional file 1: Table S1), consistent with the previous gene expression patterns. It appears that during the corolla development toward maturity, the housekeeping genes and target genes were all up-regulated, yielding significant correlations in transcript numbers among them (Table 4). Although *actin 4 *was previously shown to express steadily in mature corolla of *I. nil *[26], its homolog in the common morning glory were not expressed at a constant level during earlier floral development as desired for the relative quantification method [27-29]. Instead, cell division and expansion during corolla development necessitate coordinated expressions of the housekeeping genes *IpACTIN4 *and *IpGAPDH2 *since they encode proteins vital for all cells. When the whole genomic expression changes at cellular level, the expression levels of housekeeping genes fluctuate accordingly. This correlation can only be weakened if these genes have their own regulatory circuits regardless of the genomic expression. The evidence so far does not support the latter scenario [30,31], but points to the multifunctional roles of the reference genes [32]. In contrast, the genes on the anthocyanin pathway express in the *I. purpurea *corolla only days before floral opening. Their expressions are susceptible to both developmental and environmental changes [22].

**Table 3:** CBA estimates of expression variations of reference and target genes in developing *Ipomoea purpurea *petals across environments

Variation source	Genotype	Sample size	Gene	Mean expression (transcript copies/pg cDNA)	Coefficient of Variation
From 60 HBF* until flowering	III6D	31	*IpACTIN4*	429.8	159%
			*IpGAPDH2*	247.0	106%
			*IpDFRB-fl1*	1710.5	114%
From 90 HBF until flowering	GZKL	30	*IpACTIN4*	133.6	80%
			*IpGAPDH2*	327.1	72%
			*IpF3'H-blue*	136.1	84%
			*IpWD1-a*	8.8	67%
36 HBF among four days	II8II2	4	*IpACTIN4*	86.0	66%
			*IpGAPDH2*	45.2^a^	27%
			*IpF3'H-blue*	20.1	80%
			*IpMYB1-a*	19.4	70%
	II8SX	4	*IpACTIN4*	53.8	52%
			*IpGAPDH2*	4.0^b^	113%
			*IpCHI-fl1*	44.9	93%
	S2Y6	4	*IpACTIN4*	39.2	95%
			*IpGAPDH2*	8.1^b^	139%
			*IpANS-f*	11.8	102%
	YNSX	4	*IpACTIN4*	21.7	126%
			*IpGAPDH2*	6.2^b^	165%
			*IpF3H-1*	4.8	178%
			*Ip3GT-b*	127.0	92%
Same day at four stages	SXSX2-2	4	*IpACTIN4*	128.6	119%
			*IpGAPDH2*	258.8	124%
			*IpCHSD-us1*	585.5	137%
	SXSX2-8	4	*IpACTIN4*	105.1	41%
			*IpGAPDH2*	238.5	40%
			*IpCHSD-mex9*	474.5	65%
			*IpWD1-b*	4.2	51%

*HBF refers to hours before flowering. For genotypeII8II2, four days were 6, 11, 16 and 21 of September 2010. For genotypeSXSX2-2 &-8, four stages were at 9 am, 10 am, 11 am, and 12 pm of 28 September 2010.

^a,b ^Significant Wilcoxon two-sample test was indicated between superscripts, *P *= 0.014.

**Table 4:** Spearman's correlation coefficients between transcript copy numbers in developmental petals of *Ipomoea purpurea*

Reference genes	Target genes		
	
	*IpDFR-B*	*IpF3'H*	*IpWDR1*
*IpACTIN4*	0.575* P = 0.007, n = 31	0.374 *P *= 0.042, n = 30	**0.592** *P *= 0.006, n = 30
*IpGAPDH2*	**0.786** *P *< 0.0001, n = 31	**0.881** *P *< 0.0001, n = 30	**0.796** *P *< 0.0001, n = 30

* The significant coefficients are shown in bold (*t*-test, *P *< 0.01).

**Figure 3:**
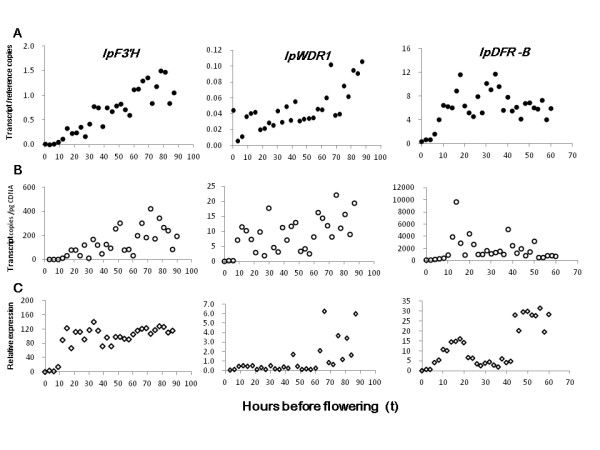
**Expression patterns of target genes during corolla development of *Ipomoea purpurea***. Three anthocyanin genes were compared. *IpF3'H *(accession number: EU032626) and *IpWDR1 *(accession number: EU032621) were both from genotype GZKL, and *IpDFR-B *(accession number: AB018438) was from III6D. (**A**) Expression pattern after normalized by the geometric means of the reference gene transcript numbers. (**B**) Direct estimates of transcript copy numbers by the CBA method, without normalization. (**C**) By the relative quantification method, with reference genes *IpACTIN4 *and *IpGAPDH2 *expressed in the same samples and calibrated at the time of flowering (t = 0).

When these target transcripts were normalized by those of the reference genes in the same cells, the practice in effect took some of the variation of cell-level transcription into account and the expression patterns of the targets became more visible (Figure 3A). There is still much space for obtaining the best reference genes that are more closely correlated with the whole genome expression. What we have presented here simply shows the effectiveness of taking the step.

### Comparing relative and absolute qPCR estimations

The largest difference between the relative quantification and the CBA methods is the interpretation of the results of qPCR. As seen in *I. purpurea*, different genotypes vary in gene expressions, for instance, between genotypes II8II2 and S2Y6 at *IpGAPDH2 *after taking developmental stage and the environment into account (Table 3). Such information might be ignored in the relative method if the control condition were taken on a separate genotype. In the absolute quantitative qPCR, results are easy to compare and interpret biologically since target genes as well as reference genes are both quantified in the same cDNA samples. The estimated transcript numbers may be readily assessed across samples and experiments.

**Figure 4:**
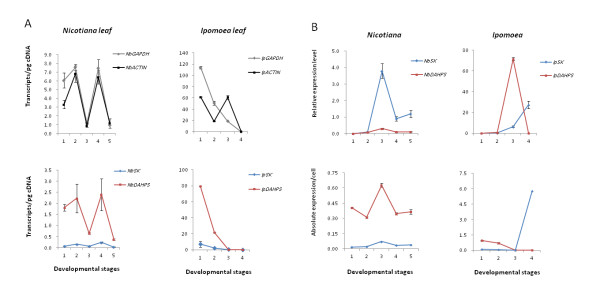
**Expression patterns of target genes through leaf developmental stages**. Five developmental stages were shown for *Nicotiana benthamiana*, and four for *Ipomoea purpurea*. The data were measured with three replicates. (**A**) The absolute quantifications of four different transcripts - two references and two targets - in *N. benthamiana *(*NbSK*: EST8653 and *NbDAHPS*: JQ256518) and *I. purpurea *(*IpSK*: JQ256515, *IpDAHPS*: JQ256519). (**B**) Results of the relative quantification vs. normalized CBA using the data in (**A**).

For making a strict comparison between the CBA and the relative methods, we applied both methods to the floral and leaf sample sets using the same reagents and chemical treatments. The gene expression pattern given by the 2^-ΔΔCt ^method appeared to be consistent with that by the CBA method (Figures 3 &4). Absolute transcript number can directly depict expression variation only when the whole genomic expression remains constant as in perhaps mature tissues. For developmental petals, a large variance of gene expression was observed in CBA method, most likely due to fluctuation of genomic expression (Figure 3B &4A). In the relative method, the expression level was calibrated to a control point; however, factors causing differential expressions between the target and reference genes could bring in biases, explaining the abrupt patterns (e.g., Figure 3C). When target gene expression was normalized by the reference gene expressions, a more continuous pattern emerged for transcript variation during floral development (Figure 3A).

Leaf samples displayed patterns present to the CBA but absent by the relative method. Random taken genes on the shikimate pathway, shikimate kinase (SK) gene and 3-deoxy-D-arabino- heptulosonate 7-phosphate synthase (DAHPS) gene, show a magnitude difference in gene expression level between leaf sample sets of *I. purpurea *and *N. benthamiana*, along with the two references (Figure 4A,Table 5). Inter-specific gene expression difference has been only recently noticed for *Drosophila *genes at a broader scale [33], and little is known of the underlying mechanism. As in *Arabidopsis thaliana*, where the expression of SK1 gene increases toward later stage of leaf development [34], we observed a similar pattern of SK gene expression in *I. purpurea *and *N. benthamiana*.

**Table 5:** CBA estimates of gene expression levels (transcript copies/pg cDNA) in the whole leaf tissues

Species	Sample size	*GAPDH2*	*ACTIN4*	*SK*	*DAHPS*
Ipomoea purpurea**	12	61.23 (1.87)*	47.18 (1.61)	3.17 (0.05)	33.67 (1.32)

*Nicotiana benthamiana*	15	5.58 (0.54)	4.36 (0.58)	0.14 (0.02)	1.78 (0.39)

*Standard errors are in the parentheses.

**Comparisons between species are significant for all four genes (all *t*-tests, *P <*0.0001).

Given that more appropriate reference genes may be identified with a wider survey, CBA-based detection of cellular level gene expressions still has room for improvement. While reliable reference genes are beneficial to both relative quantification and CBA methods, we observed a smaller variance of estimate in CBA than in the relative method when the reference expressions were taken into account (Figure 4B).

## Conclusions

Patterns of gene expression are most informative when they can be broadly compared, which is now feasible with the CBA method detailed here. As in all biological experiments, a meaningful inference relies on the degree of sensitivity of protocol, the statistical design [35], and careful handling from sample collection to data analysis [36-39]. Since a couple of nanogram of cDNA will allow one to get a reliable estimation of the transcript abundance of a target gene, gene expression in small samples may be readily assayed via the CBA method. Being widely applicable to various biological materials, the new procedure with its interpretational power represents a positive step towards a better understanding of tissue-specific or temporal expression patterns ubiquitous to biological systems.

## Methods

### Plant species and tissues

Four stages of developing leaves (1.3 - 4.3 cm in length) of *I. purpurea *and five stages of *N. benthamiana *leaves (1.8 - 5.2 cm in length) were taken from growth chambers and their RNAs were extracted using the protocol detailed below. Petals of *I. purpurea *were sampled at different developmental stages (from 90 HBF and 60 HBF) in field or at the same developmental stage (36 HBF) but in different natural environments.

In order to cover a variety of genotypes and developmental stages of gene expression and make comparisons among them, we collected corolla RNA samples from 10 genotypes of *I. purpurea*. Floral buds of III6D and GZKL were collected in time series in 2009 summer, with III6D sampled every two hours from 60 HBF (n = 31) and GZKL sampled every three hours from 87 HBF (n = 30). The sampling of the former was from 10 am of 21 September to 4 am of 25 September of 2009, while the latter was from 4 pm of 1 September to 4 am of 4 September of 2009. The rest (SXSX2-2, SXSX2-8, II8II2-d-2, II8II2-d-6, II8SX-1, S2Y6-1, SXGZ-1 and YNSX-1) were all sampled in 2010 summer. Floral buds of SXSX2-2 and SXSX2-8 were sampled from 9:00 am to 12:00 am at an hourly interval on 28th September 2010, and the others were sampled at 16:00 pm from 6th September to 21st September at a five-day interval, each at the stage of 12 HBF.

### RNA extraction and cDNA synthesis

Fresh floral buds or leaves were immediately placed in liquid nitrogen and stored at -80°Cwhen not processed immediately. RNAs were extracted using TRIzol (Invitrogen, Carlsbad, CA, USA) or TRNzol total RNA Reagent (Tiangen, Beijing, China) with the standard procedure. Following a cleaning with cold 70% alcohol, the deposit was resuspended in RNase-free ddH_2_O. Quality of the RNA solution was checked on an agarose gel and quantified approximately with a photometer. For making standard cDNA, about 3 mg RNA was added in a final volume of 50 μl buffer system including one unit of DNaseI (New England BioLabs, Ipswich, MA, USA), and incubated at 37°Cfor 10 min to digest carried over gDNA. The first-strand cDNAs were then synthesized in 20 μl volume from the treated RNAs (~3 ug) using the standard protocol of SuperScriptIII (Invitrogen) or TIANScript (Tiangen). For preparing conventional cDNA, the whole RNA was directly taken in this step without DNase treatment. After the synthesis, each synthesis reaction was added with 1.5 μl (75 units) of RNaseIf (New England BioLabs) and 2.4 μl 10× NEB buffer and incubated at 37°Cfor 20 min to clean up the remnant RNA. Protein extraction was then performed on the treated cDNA solution using equal amount of the solution of phenol (tris-saturated): chloroform: isoamyl alcohol (25:24:1). The samples were centrifuged at 4°Cand 12000 rpm for 15 min. The supernatant was extracted again using the same manner in equal volume of the solution of chloroform:isoamyl alcohol (24:1). The supernatant was then added with 10 μl 3 M NaAc (pH 5.2) and 250 μl cold alcohol, mildly mixed, and placed at -20°C for 30-60 min. After a centrifuge of 12000 rpm at 4°Cfor 20 min, the resulting deposit was washed with 70% cold alcohol and dissolved in TE solution to make (standard) cDNAs solutions.

### Nucleotide standards

We constructed DNA standard series (1000, 800, 600, 400, 200 pg/μl) from known λ DNA standard (100 μg/ml) included in Quant-iT PicoGreen dsDNA Reagent and Kits (Invitrogen) with 1 × TE solution (pH 7.5). The RNA standard series (1000, 800, 600, 400, 200 pg/μl) were prepared similarly from the rRNA standard of Quant-iT RiboGreen RNA Reagent and Kit (Invitrogen).

### Buffers and dye solutions

Two types of TE buffer (1×) were prepared. One (pH 7.5) was a direct dilution from the 20 × stock solution of the Quant-iT PicoGreen dsDNA Reagent and Kits; The other (pH 8.0) was prepared from the same stock, but added with 0.5% sodium deoxycholate (Sigma-Aldrich, St. Louis, MO, USA) and adjusted pH to be 8.0 with sodium hydroxide. A working solution (1/200) of Picogreen dye of 2 ml was made of 10 μl Picogreen stock solution and 1990 μl TE (pH 7.5) 1 × solution, while the working solution (1/200) of SYBR Green II was set up in the same dilution factor from its stock solution with 1 × TE solution (pH 8.0).

### Quantification of cDNA standard

Known concentrations of λDNA were prepared in 1 × TE (pH 8.0) solution, and included as standards in the quantification of cDNA by running the "DNA concentration measurement" module on a qPCR machine (Rotor Gene 3000, Corbett Research, Australia, http://www.corbettlifescience.com) using its Rotor-Gene 6.0.16 software (2004). Purified cDNA as described above was first measured roughly with a conventional photometer, and then made with 1 × TE (pH 8.0) solution in triplets in three concentrations (1/6, 1/10, 1/20 in our case) to be further quantified with SYBR Green II (Molecular Probe 07568). Each reaction was made in a 20 μl volume (10 μl SYBR Green II buffer +10 μl standard lambda DNA or unknown sample) and incubated at 25°Cfor 5 min, then measured 5 times at 20 s intervals in the detection channel FAM/Sybr (470/510 nm). The concentration of the cDNA was inferred from the standard linear relationship between fluorescent signal level and DNA quantity of λDNA.

### Quantification of trace DNAs and unknown cDNAs

For trace DNAs in sample cDNAs, we prepared standard λDNA series in 1 × TE (pH 7.5) solution as mentioned previously, and measured unknown cDNA samples in a 20 μl reaction volume (10 μl Picogreen buffer +10 μl standard lambda DNA or unknown) with the "DNA concentration measurement" module. The running parameters were the same as above. This step gave the estimates of the trace DNA quantities in the cDNA samples.

In the next step, the unknowns were measured in triplets with SYBR Green II following the same procedure as for the cDNA standard. When the average of triplet outputs of an unknown sample was outside the range of the standard curve, we readjusted its initial quantity to make sure that the measurements were in-range. So obtained estimate was then subtracted by the trace DNA estimate to yield the concentration of the cDNA in the unknown sample.

### Target gene standards

Taking an anthocyanin pathway gene as an example, we amplified the whole coding sequence of a bHLH gene *bh2c *(GenBank: EU032620) from floral cDNA with a high fidelity polymerase and gene-specific primers (Additional file 1: Table S2), and separated the PCR product in an agarose gel. The target band was further cleaned using TIANgel Midi purification kit (Tiangen). The column-purified DNA was then measured with λ DNA standard included in the same run. The quantified target gene standard was serially diluted to make a standard curve for the unknowns in the same qPCR run.

### Performing real-time qPCR

We started the real-time qPCR in the module of "SYBR Green I" following the standard protocol detailed in the manual of RG3000. The initial run was often tentative in terms of finding the linear range of the standard series, exploring the scope of transcript levels among unknown samples, or optimizing the amplification parameters. As the linearity of the standard series defines the range of appropriate amounts of transcripts to be detected, samples outside the range need to be readjusted to have in-range measurements as in Figure 3B. Our typical running profile was at 95°C for 20 seconds (s), then 40 cycles of 95°Cfor 5 s, 57-60°C for 10s, and 72°C for 10s using allele specific primers (Additional file 1: Table S2). The end product of the qPCR was cloned and sequenced to verify its identity. From the standard series included in the qPCR, a linear relationship between Ct and log (DNA weight) was plotted for a target transcript. Based on the relationship, the Ct value for a given sample was used to infer its corresponding amount of template. As the target gene sequence was known, the copy numbers implied in the quantity may be calculated by the molecular weight of the sequence as shown in equation (1), which led to the estimate of the copy number in the unknown sample.

### Transcript estimates of two housekeeping genes and target genes in *I. purpurea *and *N. benthamiana*

A total of 14 genes were surveyed in *I. purpurea*, including seven structural and three regulatory genes (Additional file 1: Table S3) on the anthocyanin pathway (See [22] for their accession numbers), two housekeeping gene (*IpACTIN4, IpGAPDH2*), and two genes (*IpSK *and *IpDAHPS*) on the shikimate pathway. There are four genes (two references *NbACTIN *and *NbGAPDH *and two targets *NbSK *and *NbDAHPS*) assayed for *N. benthamiana*. When expression levels of two reference genes were taken, their geometric mean [38] was utilized in the normalization of the target transcripts. All estimates were obtained via the CBA procedure, and the relative quantification of gene expression was applied according to Livak and Schmittgen [7].

### Statistical analysis

The raw data of transcript copy numbers were log-transformed to be similar to the normal distribution. For table 3, Wilcoxon two-sample test was performed manually under the null hypothesis that there is no difference between group means [40]. For additional file 1, a fixed linear regression model was estimated by REML method via the mixed procedure of SAS (9.0) (SAS Institute, Cary, NC, USA), where gene and genotype were considered fixed effects. For table 4, Spearman correlation coefficients were reported by the same software, and the significance level was set at the probability of 0.05 as the experiment-wise error rate. The standard errors estimated in Figure 4B were approximated by the delta technique using the Taylor series as previously described [41].

## Competing interests

The authors declare that they have no competing interests.

## Authors' contributions

YL conceived the study, established the protocol, analyzed the data, and wrote the manuscript. LX collected data on features of dyes and handled leaf and petal samples, both LX and JC sampled floral samples and performed subsequent qPCR experiments. All authors contributed to the initial draft and approved the final version.

## Supplementary Material

Additional file 1 **Figure S1**. Fluorescent emissions among samples with different nucleotides. **Table S1**. Fixed effects of genotype and gene on transcript copy number in developmental petals. **Table S2**. Primer sequences used in gene cloning and qPCRs. **Table S3**. Sampling scheme of the tested loci in genotypes of *I. purpurea *corolla in 2010. Click here for file
