# Supplementary material for: A novel procedure for absolute real-time quantification of gene expression patterns
Source: Plant Methods. 2012 Mar 9;8:9. doi: 10.1186/1746-4811-8-9 (PMC3323441; doi:10.1186/1746-4811-8-9)
Supplement: Additional file 1 — Figure S1. Fluorescent emissions among samples with different nucleotides. Table S1. Fixed effects of genotype and gene on transcript copy number in developmental petals. Table S2. Primer sequences used in gene cloning and qPCRs. Table S3. Sampling scheme of the tested loci in genotypes of I. purpurea corolla in 2010. [file 1746-4811-8-9-S1.DOC]

**Additional material**

Additional File 1: Figure S1 **Fluorescent emissions among samples with different nucleotides**.

Sample mixed with Picogreen was detected under 510nm. The black bars show the standard errors.

Additional File 1: Table S1 **Fixed effects of genotype and gene on transcript copy number** **in developmental petals**.

| **Effect** | **df** | **F-value** | **P-value** |
| --- | --- | --- | --- |
| Genotype | 1 | 12.86 | **0.0007** |
| Gene | 2 | 15.86 | **<0.0001** |
| Time | 3 | 7.27 | **0.0003** |
| Genotype × gene | 2 | 0.13 | 0.8745 |
| Gene × time | 6 | 0.40 | 0.8761 |

All transcript copy numbers (*IpCHS-D*, *IpACTIN4*, and *IpGAPDH2*) were log-transformed for genotypes (SXSX2-2 & -8) of *Ipomoea purpurea*. Three repeated measurements were taken on each sample. Time refers to the sampling time, and the significant tests (at the level of α = 0.05) are in bold.

**Additional File 1: Table S2 Primer sequences used in gene cloning and qPCRs.**

| **primers for gene cloning** | | |
| --- | --- | --- |
| **Gene name** | **Primer name** | **primer sequences(5'->3')** |
| *IpCHS-D* | chsd-forward | ATGGTGACCGTCGAGGAGGTC |
|  | chsd-reverse | GTCGGGCTTATGCTGGGACG |
| *IpCHI* | chi-forward | ATGTCTGCGCCGC |
|  | chi-reverse | GGCTGCAGTTATTTAGAGACGACG |
| *IpF3H* | f3h-forward | ATGACGACGGTGTCAACCTTA |
|  | f3h-reverse | CGCATATATATACTTTGTGACTTTACTGCA |
| *IpF3'H* | f3'h-forward | ATGGCTACCTTAACCTTA |
|  | f3'h-reverse | CGCTGCAGTTAATTGAGAGTATAGAGAT |
| *IpDFR-B* | dfr-forward | ATGGTGGACGGTAATCATCCTCTTC |
|  | dfr-reverse | GCGCTGCAGCTTCTCAAGCTTTTAA |
| *IpANS* | ans-forward | ATGCTGTCTACTATTACTGCAACTGTTC |
|  | ans-reverse | CGCGAAGCTTCTAGCTAATTTGATTGA |
| *Ip3GT* | 3gt-forward | ATGGGCAGTTCAGAGTGCCATG |
|  | 3gt-reverse | CGCGCTGCAGTTATCAAAAATAACCAT |
| *IpMYB1a* | myb-forward | TATGGATCCATGGTTAATTCTTCTGCAAG |
|  | myb-reverse | TATCTCGAGTTAAATGGTTGTGTCTAAAAG |
| *IpbHLH2* | bh2c-forward | GGACTCGAGCTAAAACTGAGGAATTATRCTATG |
|  | bh2c-reverse | GATGGATCCATGGCGGAAACCCCTG |
| *IpWDR1* | wd1-forward | CAAAGATGGTGAACTCAAC |
|  | wd1-reverse | CATCTCCTTACACTTTTAGC |
| *NbACTIN* | nbact-forward | GAGGATATTCAGCCACTCGTCTG |
|  | nbact-reverse | GCCTTTGCAATCCACATCTGTTGG |
| *NbGAPDH* | nbgap-forward | GTCTTTGGAATTAGGAACCCTGAAG |
|  | nbgap-reverse | ACATCAACAGTTGGAACTCTGAAGG |
| *NbDAHPS* | nbphs-forward | CAATGGCTCTTTCAAGTAGTAGCACTAC |
|  | nbphs-reverse | CACAGTTCGTGATCCACCAATGC |
| *NbSK* | nbsk-forward | ATGGAGGCTAGAGTTTCACAGAGC |
|  | nbsk-reverse | CATATGTCTCCAATTAATGGGACGAAC |
| *IpACTIN4* | ipact-forward | CGAGAAAGGGAATAACTTCCGGCGATC |
|  | ipact-reverse | CTTGTATGCCACGAGCATCTTGGATC |
| *IpGAPDH2* | ipgap-forward | GCTTTAAGCCTCCGCCATGGG |
|  | ipgap-reverse | ACGTTGGAAGCAATAAGCCCTTAAGCAG |
| *IpDAHPS* | ipphs-forward | GCAGAGCGTTACAGACATCATCG |
|  | ipphs-reverse | GACATGGTGTCATCAAACCTAAGCG |
| *IpSK* | ipsk-forward | GATCAATTGAGAGGAGTTGGAGAAGCAG |
|  | ipsk-reverse | GCATGTATATGATGGAGCTCAATGCACT |
| **primers for real-time PCR** | | |
| **Gene name** | **Primer name** | **primer sequences(5'-3')** |
| *IpCHS-D* | chsd-d2for | TGGGACGCTATGGAGGAC |
|  | chsd-d3p | GAACAACATCGAGAAATGCTTGTCGG |
| *IpCHI* | chi-450 | CGCCTCTATCTTCTTCACTC |
|  | chi-710 | CGACCGTTTGTGGAATGAC |
| *IpF3H* | f3h-370 | GGTGAAGCAGTGAAAGATTGG |
|  | f3h-580 | GTCCATTTCCACACAGGCTT |
| *IpF3'H* | f3ph-600 | GACCCAAAGGCGGAGGAG |
|  | f3ph-880 | TCCCTCTTCGCTATCCGTATCAG |
| *IpDFR-B* | dfr-760 | GCAGAAGGAAGATTCATCTGCT |
|  | dfr-1030 | GCTCTTCTTCAATTGCAGCAG |
| *IpANS* | ans-850 | GCAACGGCAAGTACAAGAG |
|  | ans-1110 | GATTGATGATCATCATCATTATCAGG |
| *Ip3GT* | 3gt-1114 | CCATTCTATGGGGATCAGCA |
|  | 3gt-1356 | AAGCAGGTGCACTAATTCTTGGAA |
| *IpMYB1a* | myb1a-540 | GCTAAGGCTGTCGTCTATG |
|  | myb1a-810 | GGTCCACATCAATCGGAAAG |
| *IpbHLH2* | bh2c-970 | GTATTCGGGTGGGAACG |
|  | bh2c-1270 | TGGAACAGGTGGAGCTG |
| *IpWDR1* | wd1-290 | GTCAATGAAGTCCGCCG |
|  | wd1-525 | TCAATTGGGTTTCCACGACG |
| *NbACTIN* | nbact-8 | TGGGTATGGGTCAGAAAGATGC |
|  | nbact-226 | TTTCACGATTAGCCTTTGGGTT |
| *NbGAPDH* | nbgap-6 | TGGAATTAGGAACCCTGAAGAA |
|  | nbgap-218 | GAGACAATGTCATATTCTGGCTTG |
| *NbDAHPS* | nbphs-134 | GGTCGAGTTTTTAGACCGCAAG |
|  | nbphs-433 | GAGAACTAGGCAGTTGGATGGTG |
| *NbSK* | nbsk-32 | TGTCGTCATGGCTTAATTCGG |
|  | nbsk-281 | TCTTCTGATTTATTCTTCAAAGTCTCG |
| *IpACTIN4* | ipact-924 | CGGTATTGCGGATAGAATGAGC |
|  | ipact-1034 | GAGCCTCCAATCCAGACACTG |
| *IpGAPDH2* | ipgap-539 | TCCACTCCATCACTGCCACCCA |
|  | ipgap-700 | ACATTCCGGTCAGTTTCCCATTAAGAG |
| *IpDAHPS* | ipphs-508 | TTCAAGGAGTTCAATGCCAATAAC |
|  | ipphs-713 | GCATCTCCATTTACGTTGTCTCC |
| *IpSK* | ipsk-6 | ATTGAGAGGAGTTGGAGAAGCAG |
|  | ipsk-240 | CTGAGAAGAACATGAAGCCGTC |

Additional File 1: Table S3 **Sampling scheme of the tested loci in genotypes of *I*. *purpurea* corolla in 2010**.

| Genotypes | *CHSD* | *CHI* | *F3H* | *F3'H* | *DFR-B* | *ANS* | *3GT* | *MYB1* | *WDR1* | *BHLH2* | *Actin4* | *GAPDH2* |
| --- | --- | --- | --- | --- | --- | --- | --- | --- | --- | --- | --- | --- |
| SXSX2-2 | √ |  |  |  |  |  |  |  |  |  | √ | √ |
| SXSX2-8 | √ |  |  |  |  |  |  |  | √ |  | √ | √ |
| II8II2 |  |  |  | √ |  |  |  | √ |  |  | √ | √ |
| II8SX |  | √ |  |  |  |  |  |  |  |  | √ | √ |
| S2Y6 |  |  |  |  |  | √ |  |  |  |  | √ | √ |
| YNSX |  |  | √ |  |  |  | √ |  |  |  | √ | √ |
| III6D |  |  |  |  | √ |  |  |  |  |  | √ | √ |
| GZKL |  |  |  | √ |  |  |  |  | √ |  | √ | √ |

“√” denotes the transcript examined within each genotype.
